# Evolution of Hemoglobin Genes in a Subterranean Rodent Species (*Lasiopodomys mandarinus*)

**DOI:** 10.3390/biology9050106

**Published:** 2020-05-20

**Authors:** Hong Sun, Kaihong Ye, Denghui Liu, Dan Pan, Shiming Gu, Zhenlong Wang

**Affiliations:** 1 School of Physical Education (Main campus), Zhengzhou University, Zhengzhou 450000, China; 18239900716@163.com; 2 School of Life Sciences, Zhengzhou University, Zhengzhou 450000, China; 18236931125@163.com (K.Y.); liuyaoru0623@163.com (D.L.); 13283864279@163.com (D.P.); gushiming70@163.com (S.G.)

**Keywords:** Mandarin vole, Brandt’s vole, hypoxia, hemoglobin gene, positive selection

## Abstract

The Mandarin vole (*Lasiopodomys mandarinus*), a typical subterranean rodent, has undergone hematological adaptations to tolerate the hypoxic/hypercapnic underground environment. Hemoglobin (Hb) genes encode respiratory proteins functioning principally in oxygen binding and transport to various tissues and organs. To investigate the evolution of α- and β-hemoglobin (Hb) in subterranean rodent species, we sequenced Hb genes of the Mandarin vole and the related aboveground Brandt’s vole (*L*. *brandti**i***). Sequencing showed that in both voles, α-globin was encoded by a cluster of five functional genes in the following linkage order: *HBZ*, *HBA-T1*, *HBQ-T1*, *HBA-T2*, and *HBQ-T2*; among these, *HBQ-T2* is a pseudogene in both voles. The β-globin gene cluster in both voles also included five functional genes in the following linkage order: *HBE*, *HBE/HBG*, *HBG*, *HBB-T1*, and *HBB-T2*. Phylogenetic analysis revealed that the Mandarin vole underwent convergent evolution with its related aboveground species (Brandt’s vole) but not with other subterranean rodent species. Selection pressure analyses revealed that α- and β-globin genes are under strong purifying selection (ω < 1), and branch-site analyses identified positive selection sites on *HBAT-T1* and *HBB-T1* in different subterranean rodent species. This suggests that the adaptive evolution of these genes enhanced the ability of Hb to store and transport oxygen in subterranean rodent species. Our findings highlight the critical roles of Hb genes in the evolution of hypoxia tolerance in subterranean rodent species.

## 1. Introduction

Subterranean rodent species spend their whole lives in complete darkness at relatively stable temperatures in hypoxic and hypercapnic underground burrow systems [1,2]. Although hypoxic and hypercapnic environments present major challenges for the survival of subterranean rodent species, previous reports have noted that *Spalax carmeli*, which is indigenous to Israel, inhabits heavy clay soil and can survive under minimal oxygen (O_2_) levels (7.2%) and maximal carbon dioxide (CO_2_) levels (6.1%) [3] and that the naked mole-rat (*Heterocephalus glaber*), which is a burrowing rodent native to parts of East Africa, can tolerate hours of extreme hypoxia and survive for 18 min under total O_2_ deprivation (anoxia) without any apparent injury [4]. The ability of subterranean rodent species to survive under conditions of high CO_2_ and low O_2_ levels without severe deleterious physiological effects or behavioral changes [4,5,6] suggests that it will be interesting to investigate the specific adaptations of mammals that live in naturally rodent species.

Mechanisms underlying hypoxia tolerance identified in different subterranean rodent species include modified blood properties, anatomical and biochemical changes in respiratory organs, and differences in the structures and functions of gene products [7,8,9]. The important adaptation of subterranean rodent species to hypoxic environments are the increased blood vessel density, increased erythrocytosis, and improved hemoglobin (Hb) capacity of O_2_ delivery and storage [6,8,10]. Hb is responsible for the transport of O_2_ from the lungs to O_2_-demanding tissues, suggesting that this protein plays an important role in the survival of subterranean rodent species under low ambient O_2_ conditions.

Hb is a heterotetramer comprising two α-chain (HbA) and two β-chain (HbB) subunits that form two semirigid dimers (α1β1 and α2β2) [11,12]. The functional adaptability of Hb genes manifest both convergent and divergent adaptive evolutionary characteristics via recombination events, unequal gene exchange, genetic conversion, and mutations that aid in long-term survival in chronically hypoxic environments [13,14,15]. For example, intraspecific allele substitution involving multiple polygenic combinations has been described in the deer mouse (*Peromyscus maniculatus*) [16]; β15Trp has been shown to change to Phe in the fetal domestic yak (*Bos grunniens*) and β135Ala to Val in the adult yak [14]. Further, a positive selection site has been reported at position144 of the HbB subunit of gymnotiform electric fish [17], and parallel β13 and β83 variants have been documented in Andean hummingbirds [18]. All of these genetic alterations increase the affinity of Hb for O_2_. Although studies have investigated hypoxia-induced physiological adaptation in subterranean rodent species with a focus on the roles of vascular endothelial growth factor (VEGF), hypoxia-inducible factor-1α (Hif-1α), erythropoietin (EPO), myoglobin, neuroglobin, and cytoglobin [8], there is limited research on the adaptive evolution of Hb in subterranean rodent species.

The Mandarin vole (*Lasiopodomys mandarinus*) is a type of subterranean rodent species capable of surviving in environments with minimal O_2_ levels (16.04%) and maximal CO_2_ levels (2.55%) [19]. In the present study, we cloned and sequenced Hb genes from the Mandarin vole and the related aboveground Brandt’s vole (*L*. *brandtii*). The main objectives of the present study were to assess the structure and function of Hb in subterranean rodent species adapted to hypoxia and to provide indirect evidence of the physiological function of Hb in subterranean rodent species to highlight the biomedical significance of this protein.

## 2. Results

### 2.1. Hb Gene Structure

**Figure 1:**
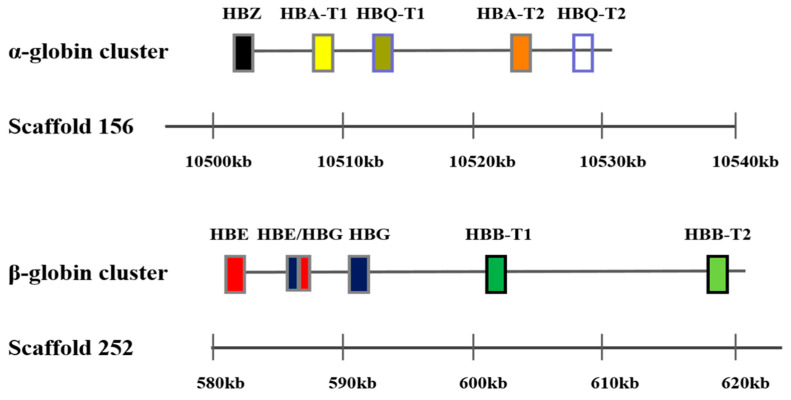
Genomic structure of the α and β-globin gene family in the Mandarin vole.

Sequencing showed that the α-globin gene cluster in the Mandarin vole included four functional globin genes: one embryonic globin gene (*HBZ*), two adult globin genes (*HBA-T1* and *HBA-T2*), and one θ globin gene (*HBQ-T1*). In the Mandarin vole, *HBQ-T2* is a pseudogene, and in terms of chromosomal structure beginning with *HBZ* and terminating with *HBQ-T2*. The β-globin gene in the Mandarin vole is a cluster of five functional globin genes in the following linkage order: one embryonic globin gene (*HBE*), one chimeric globin gene (*HBE/HBG*), one globin gene (*HBG*), and two adult globin genes (*HBB-T1* and *HBB-T2*) (Figure 1). The α- and β-globin genes in Brandt’s vole have the same structure as those in the Mandarin vole, with their amino acid compositions in the two tested species being slightly different, coding sequences and differences in the number of amino acids encoded by Hb genes in the two vole species are shown in Table 1.

### 2.2. Phylogenetic Trees

**Figure 2:**
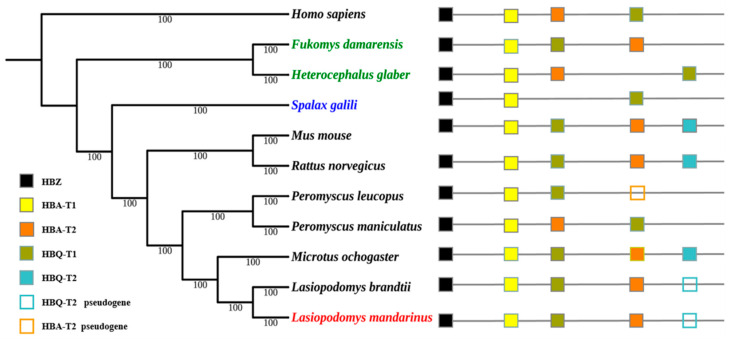
Bayesian tree of rodent species and the genomic structures of their α-globin genes. Numerals indicate Bayesian posterior probability values; colored text indicates subterranean rodent species.

**Figure 3:**
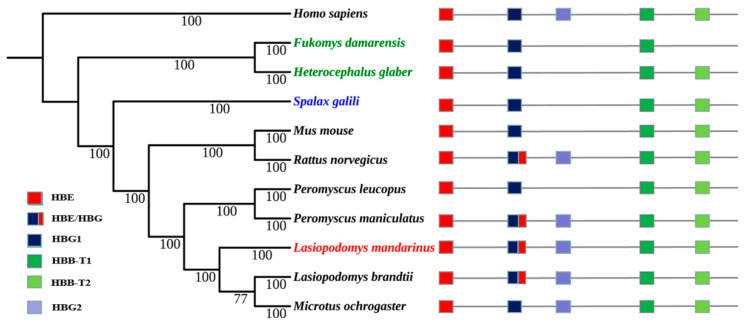
Bayesian phylogenetic tree of rodent species and the genomic structures of their β-globin genes. Numerals indicate Bayesian posterior probability values; colored text indicates subterranean rodent species.

The topology of Maximum likelihood (ML) trees was similar to that of Bayesian trees, and Bayesian trees were selected to present the final results. Bayesian trees of α- and β-globin genes were both constructed using HKY+G as the ideal model. The topology of their Bayesian trees indicated a lack of convergent evolution in subterranean rodent species, the Mandarin vole, Brandt’s vole, and the prairie vole (*Microtus ochrogaster*), all of which belong to the *Microtinae* subfamily within the *Cricetidae* family, were clustered together. The Damaraland mole-rat (*Fukomys damarensis*) and naked mole-rat, which belong to the *Bathyergidae* family, were clustered together; however, *Spalax* was located in a separate single branch (Figure 2 and Figure 3).

### 2.3. Selection Pressure Analysis

Branch models were used to calculate the ω-value of α- and β-globin genes; the estimated ω ratio of the α- and β-globin genes was <1, indicating that most Hb genes had undergone purifying selection (Supplementary Table S1). To investigate whether there are restricted positive selection sites in some specific lineages, positive selection sites were identified using the branch-site model for Hb genes (Table 2). Significant positive selection sites were found in *HBA-T1* of the *S*. *galili* lineage (12K - 0.999 *; *p* = 0.001) and **the** naked mole-rat branch (2V - 0.975 *, 21H - 0.957 *, 87L - 0.964 *, 89A - 0.981 *, 105C - 0.965 *, 124A - 0.971 *, and 134S - 0.978 *), but likelihood-ratio test (LRT) results were not significant (*p* = 0.746). Positive selection sites were also identified in *HBB-T1* of the *S*. *galili* lineage (17G - 0.966 *, 87A - 0.995 **, and 113C - 0.960 *; *p* < 0.001), naked mole-rat lineage (24V - 0.981 *, 109N - 0.929 *, 140A - 0.984 *, and 143A - 0.941 *; *p* < 0.001), and Damaraland mole-rat lineage (23E - 0.952 *, 56M - 0.992 **, 57G - 0.990 *, 70G - 0.967 *, 75G - 0.953 *, 110V - 0.983 *, 114V - 0.987 *, and 121K - 0.955 *; *p* < 0.001). However, no significant positive selection sites were identified in the other tested genes among different subterranean rodent branches.

Site models and the GARD method were used to determine whether there are recombination sites on Hb genes (Table 2). The M8 model (β and ω) showed no positive selection sites **on** *HbA-T1* but showed significant recombination sites on *HBB-T1* (6P - 1.000 **, 13T - 0.994 *, 21V - 0.960 *, 23E - 1.000 **, 24V - 0.999 **, 45S - 0.999 **, 55V -1.000 **, 57G - 0.992 **, 70G - 1.000 **, 71A - 1.000 **, 73S - 1.000 **, 77A - 0.999 **, 87A - 1.000 **, 88T - 1.000 **, 105R - 1.000 **, 110V - 0.988 *, 113C - 0.998 **, 121K - 0.996 **, 122E - 0.964 *, 126Q - 1.000 **, and 140N - 1.000 **; *p* = 0.003). To investigate the putatively selected sites with functional significance, we removed the filtered recombinant sites and mapped the remaining positive selection sites onto secondary structures. We found that the most positive selection sites were located in different secondary domains (Table 3). For example, the positive selection sites on *HBA-T1* were located in the A, B, and F helical regions and those on *HBB-T1* were located in the A, E, G, and H helical regions. However, the 2V site on *HBA-T1* was not located in a secondary domain but in the region before the A helical region.

## 3. Discussion

The performance of the hematological system of subterranean rodent species is well established. Hb plays important roles in increased O_2_ transport in response to hypoxia [12,16]. Hb gene structure and adaptive evolution have been extensively studied in species living in hypoxic environments at high altitudes and in deep ocean [20,21]. However, the Hb gene structure and evolution in subterranean rodent species remains comparatively unclear. Hence, the objective of the present study was to elucidate the evolutionary adaptation of Hb genes in subterranean rodent species to hypoxic environments.

The organization of the α- and β-globin gene clusters in the Mandarin vole parallels that in Brandt’s vole, i.e., the structure and number of functional copies of the β-globin gene clusters in both voles are similar, as documented in the deer mouse and prairie vole [22]. The structure and number of functional copies of the α-globin gene cluster were consistent between the two tested voles but differed in other rodent species. Therefore, Hb polymorphism exists among different rodent species, but no significant differences were observed in this regard between the two tested voles despite their different living environments.

Phylogenetic reconstruction of Hb genes revealed that the Mandarin vole did not undergo convergent evolution with other subterranean *Spalax*, the naked mole-rat, or the Damaraland mole-rat, but it underwent convergent evolution with the related aboveground Brandt’s vole and prairie vole. The latter two species and the Mandarin vole belong to the *Microtinae* subfamily, and their phylogeny has been shown to follow that of traditional rodent taxonomic groups [23,24]; therefore, we hypothesized that Hb genes in the Mandarin vole underwent conservative evolution.

Determination of ω ratios revealed that all Hb genes have undergone purifying selection, indicating that the Hb protein has an important function in O_2_ transport. However, the results of our analyses support the hypothesis that *HBA-T1* and *HBB-T1* in different subterranean rodent species have been subjected to positive selection, suggesting the evolutionary optimization of functionality under different environmental conditions. For example, in *HBA-*T1, amino acid substitutions in *S*. *galili* were located at a proximal binding site situated near or within the glycosylation and metal-binding domains responsible for cotranslational and post-translational protein modifications and O_2_ binding, respectively [21]. In naked mole-rat, substitution of residues 87 and 89 was close to the proximal histidine, which is implicated in the iron–proximal histidine linkage and seems to be essential in the maintenance of Hb–O_2_ binding [17]. Further, the substitution of residue 124 in the H helical region may change the spacing of hydrogen bonds [25], thereby affecting the molecular structure of proteins.

Furthermore, positive selection sites are located in the A, E, G, and H helical regions of *HBB-T1* in *S*. *galili*, the naked mole-rat, and the Damaraland mole-rat, and they may be essential for homology-based structural models [12]. Site 143 in the H helical region of *HBB-T1* in the Damaraland mole-rat is adjacent to β-147 histidine. A previous study showed that His-HC3 (147) of β-Hb in the common carp (*Cyprinus carpio*) plays a key role in the root effect, which is associated with non-cooperative O_2_ binding and decreased O_2_ affinity [17]. Another study also demonstrated that β-globin underwent adaptive evolution in the A helical region in the pika; therefore, selected 17 sites in *S*. *galili* in their study may have important Hb-associated functions in O_2_ binding [25]. Other positive selection sites were localized to residues of postulated regions and may affect these functions [26,27,28]. Thus, we hypothesized that positive selection sites in these two genes are involved in modulating Hb–O_2_ binding and we demonstrated adaptability to different hypoxia-associated elevational zones in two subterranean species.

It is worth mentioning that there were no noteworthy sites on Hb genes in the vole mandarin and Brandt’s vole. This is possibly because there is no O_2_ deficiency in the living environment of Brandt’s vole as it is an aboveground rodent. Although the Mandarin vole is a subterranean rodent, the O_2_ level in their burrow system is relatively low compared with that in the environments of other subterranean species [3,4,19]. For example, *S*. *galili* can survive under conditions of minimal O_2_ levels (12.7%) and the naked mole-rat can tolerate hours of extreme hypoxia and survive 18 min of total O_2_ deprivation (anoxia) without any apparent injury. The lowest O_2_ level measured in high-water-content soil of a burrow system of the Mandarin vole was only 16.04%, but the physiological characteristics of blood in this species including increased HIF-1α and VEGF levels and blood vessel density and decreased EPO level under hypoxia, indicating adaption to hypoxia.

## 4. Materials and Methods

### 4.1. Animals

Juvenile Mandarin voles were captured on a farmland located in Xinzheng, Henan Province, China (34°52’ N, 113°85’ E). Brandt’s vole were obtained from a prairie in Xilinhot, Inner Mongolia Autonomous Region, China (43°02′–44°52′ N, 115°13′–117°06′ E). The voles were individually housed in polycarbonate cages measuring 37 × 26 × 17 cm^3^ and kept at 20 °C–23 °C. Food and water were provided *ad libitum* (Laboratory Animal Center of Henan Province, China). Carrots were supplemented as an accessory. Both species were raised under a 14:10-h light:dark photoperiod (illumination time, 8:00–22:00 h; light intensity, 200 Lux). At least three adult voles (35 ± 5 g) were then sacrificed by cervical dislocation after induction of anesthesia with 3% pentobarbital sodium; brain tissues (0.55 ± 0.05 g) were obtained and immediately preserved in liquid nitrogen for further research. The procedures performed in this study were approved by the Animal Care and Use Committee of Zhengzhou University and performed in accordance with the Guide for the Care and Use of Laboratory Animals of China.

### 4.2. DNA Extraction, Amplification, and Sequencing

Total DNA was extracted from the brain tissues using the TIANamp Genomic DNA Kit [TIANGEN Biotech (Beijing) Co., Ltd., Beijing, China]. DNA quality was analyzed by agarose gel electrophoresis; DNA concentration was assessed using the NanoDrop ND-1000 spectrophotometer (NanoDrop Technologies, LLC, Wilmington, DE, USA). DNA used for amplification of Hb genes had integral bands, and the A260/A280 or A260/A230 ratios ranged from 1.9 to 2.1.

Hb genes were amplified by polymerase chain reaction (PCR) using primers designed with Oligo 7.0 (https://www.oligo.net\) with reference to sequences from closely related species (Table 4). PCR products were separated by electrophoresis using 0.1% agarose gels. Single-purpose fragment DNA strips were cut out from agarose gels, purified, and recovered using the QIAquick Gel Extraction Kit (Beijing ComWin Biotech Co., Ltd., Beijing, China). Complete sequences were inserted into the pMD19-T vector (Takara Bio, Inc., Shiga, Japan). After bacterial selection and colony PCR verification, the Sanger method was performed to sequence the PCR products using GENEWIZ (Suzhou, China). After combining the genomic information of the Mandarin vole, the complete Hb gene structure was diagramed.

### 4.3. Data Analysis

The Hb gene coding nucleotide sequences of other species were downloaded from the GenBank database (https://www.ncbi.nlm.nih.gov/genbank/) and aligned using the Basic Local Alignment Search Tool (http://www.ncbi.nlm.nih.gov). The sequence of each gene was aligned using the MUSCLE algorithm. α-globin/β-globin gene clusters were combined using SequenceMatrix 1.7.8 for the construction of phylogenetic trees [29]. Their Bayesian trees were constructed using MrBayes 3.2.6 [30,31], and the best-fit model for the MrBayes tree was selected using Modeltest. Finally, four Markov chains were run for 2 × 10^6^ generations and sampled every 1000 generations for Bayesian tree construction. ML trees for the best nucleotide models were constructed using MEGA × 10.1, and confidence levels of tree topologies were assessed using 1000 bootstrap replicates. 

We determined selection pressure for each Hb subunit gene using CODEML, a part of PAML 4.7 [32]. First, we used branch models to calculate the ω-value (dN/dS = nonsynonymous/synonymous) of Hb genes, with ω < 1, ω = 1, and ω > 1 indicating purifying, neutral, and positive selection, respectively. We used the one-ratio model that enforces the same ω ratio for all lineages and compared it with ω = 1 (i.e., ω = 1 for all lineages) to check the selection pressures of the two genes.

Second, we used a branch-site model (Model A, A null) to analyze positive selection sites on specific branches of each gene. Then, in PAML, we performed LR**T** to determine the differences in log-likelihood values between the two nested models using χ2 distribution, with an aim to determine whether the differences were significant by comparing the nested models. Finally, site models M7 and M8 were used to compute recombination sites in all assessed species [33], and the GARD method within Data Monkey (https://www.datamonkey.org/) was performed to identify the number and location of breakpoints and sequences involved in putative recombination events in different genes [34].

## 5. Conclusions

Hb genes are evolutionarily conserved and play important functions in O_2_ storage and transport in subterranean rodent species. Elucidation of the evolution of Hb genes offers a valuable framework for the identification and study of the mechanism underlying adaptation to hypoxic environments in subterranean rodent species. Further functional experiments including functional characterization of Hb proteins are expected to completely reveal these mechanisms. 

## Figures and Tables

**Table 1:** Lengths and accession numbers of Hb genes and differences in the number of amino acids encoded by Hb genes in the Mandarin and Brandt’s vole.

Gene		Mandarin vole		Brandts’ Vole		Number of Amino Acid Differences
		Length	Acession Number	Length	Acession Number	
α-globin cluster	*HBZ*	1685 bp	MN402680	1685 bp	MN402679	0
	*HBA-T1*	700 bp	MN402682	700 bp	MN402681	1
	*HBA-T2*	700 bp	MN402684	700 bp	MN402683	2
	*HBQ-T1*	647 bp	MN402686	647 bp	MN402685	1
	*HBQ-T2*	647 bp	MN402688	647 bp	MN402687	3
β-globin cluster	*HBE*	1307 bp	MN402690	1307 bp	MN402689	0
	*HBE/HBG*	1664 bp	MN402692	1664 bp	MN402691	0
	*HBG*	1393 bp	MN402694	1393 bp	MN402693	0
	*HBB-T1*	1141 bp	MN402696	1141 bp	MN402695	1
	*HBB-T2*	1141 bp	MN402698	1141 bp	MN402697	1

**Table 2:** Selection pressure analyses of Hb genes **in rodent species** using branch-site and site models.

Gene	Modle	-lnL	Parameter Estimates	Parameter Estimates	Positively Selected Sites	*p* Value
Branch—site Model						
HBA-T1	Branch: *Lasiopodomys mandarinus*					
	Null	2021.164	ω0 = 0.066, ω1 = 1, ω2 = 1			
	Model A	2021.164	ω0 = 0.066, ω1 = 1, ω2 = 1	Model A vs. Null	NA	1.000
	Branch: *Spalax galili*					
	Null	2021.534	ω0 = 0.068, ω1 = 1, ω2 = 0.065			
	Model A	2016.368	ω0 = 0.068, ω1 = 92.003, ω2 = 92.003	Model A vs. Null	12K - 0.999 *	0.001
	Branch: *Heterocephalus glaber*					
	Null	2004.606	ω0 = 0.032, ω1 = 1, ω2 = 1			
	Model A	2004.554	ω0 = 0.033, ω1 = 1.166, ω2 = 1.166	Model A vs. Null	2V - 0.975 *, 21H - 0.957 *, 87L - 0.964 *, 89A - 0.981 *, 105C - 0.965 *, 124A - 0.971 *	0.746
	Branch: *Fukomys damarensis*					
	Null	2021.577	ω0 = 0.067, ω1 = 1, ω2 = 1			
	Model A	2021.577	ω0 = 0.067, ω1 = 1, ω2 = 1	Model A vs. Null	NA	1.000
HBB-T1	Branch: *Lasiopodomys mandarinus*					
	Null	1578.455	ω0 = 0.002, ω1 = 1, ω2 = 1			
	Model A	1578.455	ω0 = 0.002, ω1 = 3.742, ω2 = 3.742	Model A vs. Null	NA	1.000
	Branch: *Spalax galili*					
	Null	1578.455	ω0 = 0.002, ω1 = 1, ω2 = 1			
	Model A	1565.826	ω0 = 0, ω1 = 999, ω2 = 999	Model A vs. Null	17G - 0.966 *, 87A - 0.995 **, 113C - 0.960 *	0.000
	Branch: *Heterocephalus glaber*					
	Null	1578.230	ω0 = 0, ω1 = 1, ω2 = 1			
	Model A	1568.434	ω0 = 0, ω1 = 999, ω2 = 999	Model A vs. Null	24V - 0.981 *, 109N - 0.929 *, 140A - 0.984 *, 143A - 0.941 *	0.000
	Branch: *Fukomys damarensis*					
	Null	1577.920	ω0 = 0, ω1 = 1, ω2 = 1			
	Model A	1567.132	ω0 = 0, ω1 = 999, ω2 = 999	Model A vs. Null	23 - 0.952 *, 56M - 0.992 **, 57G - 0.990 *, 70G - 0.967 *, 75G - 0.953 *, 110V - 0.983 *,114V - 0.987 *, 121K - 0.955 *	0.000
Site Model						
HBB-T1	M7: β	2558.967	p = 0.15303, q = 1.97705			
	M8: β and ω	2553.328	p0 = 0.94925, p1 = 0.05075, p = 0.17988, ω = 2.30313, q = 0.47855	M8 v sM7	6P - 1.000 **, 13T - 0.994 *, 21V - 0.960 *, 23E - 1.000 **, 24V - 0.999 **, 45S - 0.999 **, 55V - 1.000 **, 57G - 0.992 **, 70G - 1.000 **, 71A - 1.000 **, 73S - 1.000 **, 77A - 0.999 **, 87A - 1.000 **, 88T - 1.000 **, 105R - 1.000 **, 110V - 0.988 *, 113C - 0.998 **, 121K - 0.996 **, 122E - 0.964 *, 126Q - 1.000 **, 140N - 1.000 **	0.003

**Table 3:** Detected positive selection sites in different helical regions.

Gene	Position	*Spalax galili*	*Heterocephalus glaber*	*Fukomys damarensis*	Secondary Doamin
HBA-T1	2V		V→S		
	12K	K→R			A-Helix
	21H		H→S		B-Helix
	87L		L→K		F-Helix
	89A		A→S		F-Helix
	124A		A→L		H-Helix
HBB-T1	17G	G→S			A-Helix
	56M			M→V	D-Helix
	75G			G→S	E-Helix
	109N		N→D		G-Helix
	114V			V→A	G-Helix
	143A			H→G	H-Helix

**Table 4:** Primers used for the amplification of Hb genes in the present study.

Gene	Forward Primer	Reverse Primer
*HBZ*	GTCACCCTGTCTGATAACAAGC	TAACGGGTCCTAAAGACTTACCAG
	TTACAGGCAGTTGTGAGCTACCAT	ACAGGACCAGTTATTTCCGTCT
*HBA-T1*	GCCTTCTCTGCACAGGACTCT	CCCAGGCTTCCCCGTGTCA
*HBA-T2*	AACCACCCTAGTCAGCCAATGAGG	TCCAGAAGACGCCCTGAAGCTC
*HBQ-T1*	ACAGAAACAGGCTTCATATCCTC	AGGCTGAGTTACACAAGACCA
	ACTACTCGAGGGAAAGTTACGC	GCCTCTTGGAACCTTGCTT
*HBE*	CTGGCCCTCTCATAACCTG	CCAGTCCAGTACTCATGTGC
*HBE/HBG*	TGTCTTGCCCAGCCTCTCTTGACC	ATTAAGGCTGAGGAAGACAACCCA
	AAAAGAATGAAAGTTAAGAGCGTGA	CCAGGAGCTATAAGAGAAAACACAA
*HBG*	CCTGCTTGACACTATCTTACTGG	CCAGTCCAGTACTCATGTGC
*HBB-T1*	GTTGCTCCTCACACTTGCT	TAGTCAGAAGAAAGATGCCCCA
*HBB-T2*	CTAAGTCAGTGCCATAGCC	AGGTCTTCATTATTTAGCCCAA

## Supplementary Materials

Supplementary Materials can be found at https://www.mdpi.com/2079-7737/9/5/106/s1. Table S1: Likelihood-ratio test of branch models examining Hb genes.

## Abbreviations

Hb	Hemoglobin
O_2_	Oxygen
CO_2_	Carbon dioxide
ML	Maximum likelihood
LRT	Likelihood-ratio test
VEGF	Vascular endothelial growth factor
Hif-1α	Hypoxia-inducible factor-1α
EPO	Erythropoietin
