# Supplementary material for: Evolution of Hemoglobin Genes in a Subterranean Rodent Species (Lasiopodomys mandarinus)
Source: Biology (Basel). 2020 May 20;9(5):106. doi: 10.3390/biology9050106 (PMC7284791; doi:10.3390/biology9050106)
Supplement: Supplementary file 1 [file biology-09-00106-s001.pdf]

Supplement Table S1. Likelihood ratio tests of branch models examining the Hb genes

Gene	Model	-lnL	ω		*P*
**HBZ**	A:One-ratio	1812.482	ω = 0.1475		
	B:Omega = 1	1796.477	ω = 1	A vs B	< 0.01
**HBA1**	A:One-ratio	1841.054	ω =0.1612		
	B:Omega = 1	1821.828	ω=1	A vs B	< 0.01
**HBA2**	A:One-ratio	2349.633	ω = 0.1693		
	B:Omega = 1	2332.835	ω = 1	A vs B	< 0.01
**HBQ**	A:One-ratio	2087.728	ω = 0.1539		
	B:Omega = 1	2071.518	ω = 1	A vs B	< 0.01
**HBE**	A:One-ratio	1553.679	ω = 0.1353		< 0.01
	B:Omega = 1	1532.768	ω = 1	A vs B	
**HBB1**	A:One-ratio	1918.245	ω = 0.2345		
	B:Omega = 1	1864.864	ω = 1	A vs B	< 0.01
**HBB2**	A:One-ratio	1920.246	ω = 0.2696		
	B:Omega = 1	1910.104	ω = 1	A vs B	< 0.01
**HBG**	A:One-ratio	1829.056	ω = 0.2331		
	B:Omega = 1	1824.488	ω = 1	A v sB	< 0.01
